# ^1^H, ^13^C and ^15^N chemical shift assignment of the stem-loop 5a from the 5′-UTR of SARS-CoV-2

**DOI:** 10.1007/s12104-021-10007-w

**Published:** 2021-01-23

**Authors:** Robbin Schnieders, Stephen A. Peter, Elnaz Banijamali, Magdalena Riad, Nadide Altincekic, Jasleen Kaur Bains, Betül Ceylan, Boris Fürtig, J. Tassilo Grün, Martin Hengesbach, Katharina F. Hohmann, Daniel Hymon, Bozana Knezic, Andreas Oxenfarth, Katja Petzold, Nusrat S. Qureshi, Christian Richter, Judith Schlagnitweit, Andreas Schlundt, Harald Schwalbe, Elke Stirnal, Alexey Sudakov, Jennifer Vögele, Anna Wacker, Julia E. Weigand, Julia Wirmer-Bartoschek, Jens Wöhnert

**Affiliations:** 1 Department of Biology, Technical University of Darmstadt, Schnittspahnstr. 10, 64287 Darmstadt, Germany; 2 Institute for Organic Chemistry and Chemical Biology, Johann Wolfgang Goethe-University Frankfurt, Max-von-Laue-Str. 7, 60438 Frankfurt/M., Germany; 3 Institute for Molecular Biosciences, Johann Wolfgang Goethe-University Frankfurt, Max-von-Laue-Str. 9, 60438 Frankfurt/M., Germany; 4 Center for Biomolecular Magnetic Resonance (BMRZ), Johann Wolfgang Goethe-University Frankfurt, Max-von-Laue-Str. 9, 60438 Frankfurt/M., Germany; 5 Present Address: EMBL Heidelberg, Meyerhofstraße 1, 69117 Heidelberg, Germany; 6 Department of Medical Biochemistry and Biophysics, Karolinska Institute, Biomedicum, Solnavägen 9, 17177 Stockholm, Sweden

**Keywords:** SARS-CoV-2, 5′-UTR, SL5a, Solution NMR spectroscopy, COVID19-NMR

## Abstract

The SARS-CoV-2 (SCoV-2) virus is the causative agent of the ongoing COVID-19 pandemic. It contains a positive sense single-stranded RNA genome and belongs to the genus of Betacoronaviruses. The 5′- and 3′-genomic ends of the 30 kb SCoV-2 genome are potential antiviral drug targets. Major parts of these sequences are highly conserved among Betacoronaviruses and contain *cis*-acting RNA elements that affect RNA translation and replication. The 31 nucleotide (nt) long highly conserved stem-loop 5a (SL5a) is located within the 5′-untranslated region (5′-UTR) important for viral replication. SL5a features a U-rich asymmetric bulge and is capped with a 5′-UUUCGU-3′ hexaloop, which is also found in stem-loop 5b (SL5b). We herein report the extensive ^1^H, ^13^C and ^15^N resonance assignment of SL5a as basis for in-depth structural studies by solution NMR spectroscopy.

**Supplementary Information:**

The online version contains supplementary material available at 10.1007/s12104-021-10007-w.

## Biological context

SCoV-2 is a member of the Betacoronavirus family and contains a large single-stranded (+) RNA genome with a length of approx. 30,000 nucleotides (nts) (Hu et al. 2020; V’kovski et al. 2020). The RNA genome of the virus not only contains the coding regions for the viral proteins, but also extended and highly structured 5′- and 3′-UTRs, as well as internal structured RNA elements with important functional roles in genome replication, transcription of subgenomic (sg) mRNAs and the balanced translation of viral proteins (Madhugiri et al. 2016; Kelly et al. 2020; Tidu et al. 2020). While the development of antiviral therapeutics against COVID-19 is primarily focused on the viral proteins, the highly structured RNA elements provide an extensive reservoir of additional drug targets to be exploited. The architecture of the RNA genome of SCoV2 and related viruses has so far been investigated mainly by sequence-based computational predictions and by chemical probing approaches in vitro and in vivo (e.g. Manfredonia et al. 2020; Rangan et al. 2020). Although structural probing methods have been established to map RNA-small molecule interactions even in cells (Martin et al. 2019), these tools are unable to define the tertiary structure and dynamics of the RNA-elements in the SCoV-2 genome with sufficiently high resolution to enable structure-based drug design by virtual screening.

While the sequences of the individual structural elements vary between different Coronaviruses, their ubiquitous presence and highly conserved secondary structures suggest that these elements are critically important for viral viability and pathogenesis (reviewed in Madhugiri et al. 2016). One example of such an important structure is stem-loop 5 (SL5). SL5 is structurally conserved in the genomes of Alpha- and Betacoronaviruses and has been shown to be crucial for efficient viral replication (Chen and Olsthoorn 2010; Guan et al. 2011).

In SCoV-2, SL5 consists of four helices including nts 149–297 of the 5′-UTR and the first 29 nts of the Nsp1 coding region (Suppl. Figure 1A). Sub-elements are joined to the SL5 basal stem by a four-helix junction. These sub-elements are termed SLs 5a, 5b and 5c. SL5a consists of 31 nucleotides and represents the largest of the three stem-loops. Intriguingly, the apical loop sequences of SL5a and SL5b are identical (5′-UUUCGU-3′) and belong to the 5′-UUYCGU-3′ motif, which is also found in Alphacoronaviruses. This high level of sequence conservation suggests functional importance, e.g. in viral packaging (Masters 2019). Thus, we have recently obtained secondary structure models of SL5a-c and the basal stem segment of SL5 based on initial ^1^H and ^15^N assignments (Wacker et al. 2020). In order to characterize SL5a further, we provide here a near complete ^1^H, ^13^C and ^15^N chemical shift assignment.

## Methods and experiments

### Sample preparation

RNA synthesis for NMR experiments: For DNA template production, the sequence of SL5a together with the T7 promoter was generated by hybridization of complementary oligonucleotides and introduced into the *EcoR*I and *Nco*I sites of an HDV ribozyme encoding plasmid (Schürer et al. 2002), based on the pSP64 vector (Promega). RNAs were transcribed as HDV ribozyme fusions to obtain a homogeneous 3′-end. The recombinant vector pHDV-5_SL5a was transformed and amplified in the *Escherichia coli* strain DH5α. Plasmid-DNA was purified using a large scale DNA isolation kit (Gigaprep; Qiagen) according to the manufacturer’s instructions and linearized with *Hind*III prior to in-vitro transcription using the T7 RNA polymerase P266L mutant, which was prepared as described in (Guillerez et al. 2005). 15 ml transcription reactions [20 mM DTT, 2 mM spermidine, 200 ng/µl template, 200 mM Tris/glutamate (pH 8.1), 40 mM Mg(OAc)_2_, 12 mM NTPs, 32 µg/ml T7 RNA Polymerase, 20% DMSO] were performed to obtain sufficient amounts of SL5a RNA (5′-pppGGGCUGCUUACGGUUUCGUCCGUGUUGCAGCCC-3′). Preparative transcription reactions (6 h at 37 °C and 70 rpm) were terminated by addition of 150 mM EDTA. SL5a RNA was purified as follows: RNAs were precipitated with one sample volume of ice-cold 2-propanol. RNA fragments were separated on 15% denaturing polyacrylamide (PAA) gels and visualized by UV shadowing at 254 nm. SL5a RNA was excised from the gel and eluted using the following protocol: The gel fragments were granulated in two gel volumes 0.3 M NaOAc solution, incubated for 30 min at − 80 °C, followed by 15 min at 65 °C. The RNA was further eluted from gel fragments overnight by passive diffusion into 0.3 M NaOAc, precipitated with EtOH and desalted via PD10 columns (GE Healthcare). Residual PAA was removed by reversed-phase HPLC using a Kromasil RP 18 column and a gradient of 0–40% 0.1 M acetonitrile/triethylammonium acetate. After freeze-drying of RNA-containing fractions and cation exchange by LiClO_4_ precipitation (2% in acetone), the RNA was folded in water by heating to 80 °C followed by rapid cooling on ice. Buffer exchange to NMR buffer (25 mM potassium phosphate buffer, pH 6.2, 50 mM potassium chloride) was performed using Vivaspin centrifugal concentrators (2 kDa molecular weight cut-off). Purity of SL5a was verified by denaturing PAA gel electrophoresis and homogenous folding was monitored by native PAA gel electrophoresis, loading the same RNA concentration as used in NMR experiments.

Using this protocol, two NMR samples of SL5a, an 810 µM uniformly ^15^N- and a 680 µM uniformly ^13^C,^15^N-labeled sample, were prepared and used for the assignment presented herein.

### NMR experiments

NMR experiments using the ^15^N-labeled RNA were carried out at the Karolinska Institute (KI) using a Bruker AVANCEIII 600 MHz NMR spectrometer equipped with a 5 mm, z-axis gradient ^1^H [^13^C, ^15^N, ^31^P]-QCI cryogenic probe. All NMR experiments with the ^13^C,^15^N-labeled RNA were conducted at the Center for Biomolecular Magnetic Resonance (BMRZ) at the Goethe University (GU) Frankfurt using Bruker AVIIIHD NMR spectrometers from 600 to 800 MHz, which are equipped with the following cryogenic probes: 5 mm, z-axis gradient ^1^H [^13^C,^31^P]-TCI cryogenic probe (600 MHz), 5 mm, z-axis gradient ^1^H [^13^C, ^15^N, ^31^P]-QCI cryogenic probe (700 MHz) and ^13^C-optimized 5 mm, z-axis gradient ^13^C, ^15^N [^1^H]-TXO cryogenic probe (800 MHz).

At BMRZ and KI, experiments were performed at 298 K if not indicated otherwise. NMR spectra were processed and analyzed using Topspin versions 4.0.8 (GU) and 3.6.2 (KI). The chemical shift assignment was conducted using Sparky (Lee et al. 2015). NMR data were managed and archived using the platform LOGS (2020, version 2.1.54, Signals GmbH & Co KG, www.logs.repository.com). ^1^H chemical shifts were referenced externally to DSS, and ^13^C and ^15^N chemical shifts were indirectly referenced from the ^1^H chemical shift as described earlier (Wishart et al. 1995).

**Fig. 1:**
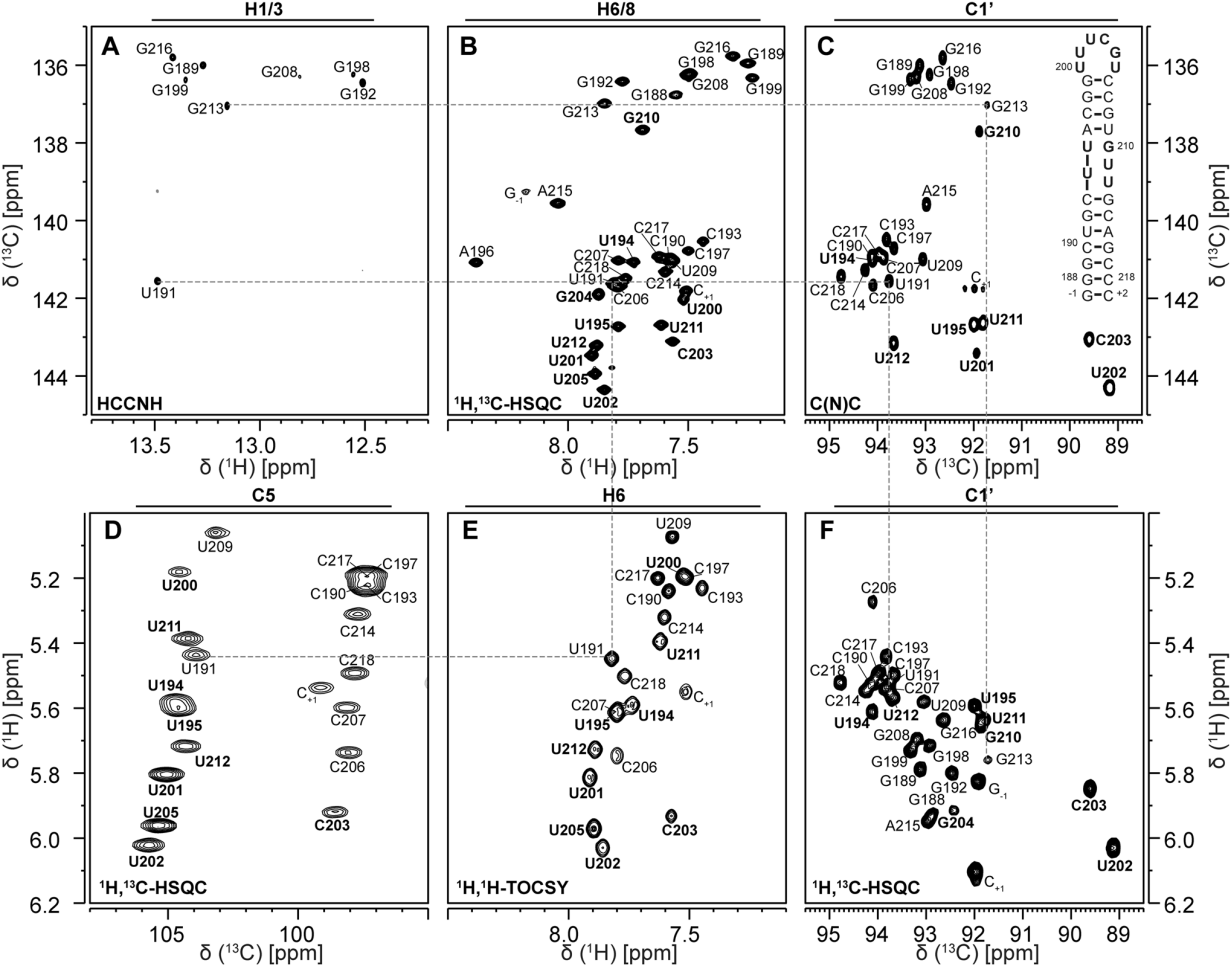
Resonance assignment of aromatic protons and carbons and the linkage to the ribose. **a** HCCNH experiment correlating the imino protons of guanosines and uridines to the corresponding intranucleobase C8 and C6 resonances, respectively. **b** ^1^H,^13^C-HSQC spectrum showing the aromatic H6/8–C6/8 correlations. **c** 2D plane of the ^13^C-detected CNC-experiment correlating C6/8 to C1′. **d** Transposed ^1^H,^13^C-HSQC spectrum showing the H5–C5 correlations for uridines and cytidines. **e** ^1^H,^1^H-TOCSY spectrum linking H5 and H6 in pyrimidines. **f** Transposed ^1^H,^13^C-HSQC spectrum of the H1′–C1′ region. Panel **c** further shows the secondary structure of SL5a with genomic numbering. Positive contours are given in black, negative contours are held in red. Experimental details are given in Table 1. Exemplary connections between the displayed spectra are demonstrated with the gray dashed lines for residues G213 and U191. Assignments of the asymmetric bulge and the apical loop are highlighted with bold font

We have previously reported the imino and cytidine amino resonance assignment of SL5a (Wacker et al. 2020) that allowed us to determine the base pairing in this RNA element. The location of stable base pairs is confirmed by through space ^2h^J_NN_ coupling constants (Dingley et al. 2008) reported in Suppl. Table S1. These assignments were available from experiments conducted on a ^15^N-labeled RNA sample and provided starting points of the aromatic proton resonance assignment using ^1^H,^1^H-NOESY (Tables 1 I, 2 I) and (H)C(CCN)H (Tables 1 IV, 2 V) experiments linking the imino proton resonances to the aromatic protons and carbons (Fig. 1a and b). The remaining H6/8–C6/8 resonances in the aromatic ^1^H,^13^C-HSQC spectrum (Tables 1 II, 2 III) were assigned using a 3D ^13^C-NOESY-HSQC experiment (Table 1 VII), which was selective for the aromatic region. Cytidine and uridine C5-H5 resonances were assigned using ^1^H,^1^H-TOCSY (Table 1 VI, Fig. 1e) and ^1^H,^13^C-HSQC spectra (Table 1 III, Fig. 1d). Furthermore, quaternary carbon atoms were assigned using an HNCO type experiment (Table 2 IV) and the TROSY relayed HCCH-COSY experiment (Table 1 VIII). The ^13^C-detected 3D CNC spectrum (Table 1 V, Fig. 1c) linked the aromatic carbons to the anomeric C1′ resonances, where the nitrogen dimension aided in distinguishing between purine and pyrimidine nucleotides as well as between uridines and cytidines. Also, by correlating C6/8 to C1′, resonance overlap is minimized given the broader signal distribution in the carbon as opposed to the respective proton dimensions. Based on C1′ resonances obtained from the CNC spectrum and from sequential assignment in the NOESY spectra, H1′–C1′ correlations were assigned in the ^1^H,^13^C-HSQC spectrum (Table 1 III, Fig. 1f). A continuous sequential walk of H1′-to-H6/H8 was possible for both helices (Fig. 1c). The H1′–C1′ assignment was further confirmed with a 3D ^13^C-NOESY-HSQC experiment (Table 1 IX), which was selective for the C1′ resonances. Using two different 3D HCCH TOCSY experiments (Table 1 X, XI and XII), the remaining ribose carbon resonances C2′–C5′ were assigned. The two experiments differed in the TOCSY mixing time such that with a short mixing time of 6 ms, C2′ and C3′ resonances could be distinguished by intensity differences, while with a long mixing time of 18 ms also C4′ and C5′ carbons were correlated to the C1′ resonances.

**Table 1:** List of NMR experiments conducted at KI and BMRZ at 298 K

#	NMR experiment	Experimental parameters	Characteristic parameters
I	^*1*^*H,*^*1*^*H-NOESY*^*KI*^ Jump-return water suppression	A Aromatics: 600 MHz, ns: 64, sw(f2): 21.0 ppm, sw(f1): 11.9 ppm, aq(f2): 81 ms, aq(f1): 56 ms, o1(^1^H): 4.7 ppm, o3(^15^N): 153 ppm, rel. delay: 1.5 s, time: 25 h B Iminos: 600 MHz, ns: 128, sw(f2): 21.0 ppm, sw(f1): 11.9 ppm, aq(f2): 81 ms, aq(f1): 25 ms, o1(^1^H): 4.7 ppm, o3(^15^N): 153 ppm, rel. delay: 1.0 s, time: 16 h	A NOE mixing time 150 ms, JR-delay 167 µs B NOE mixing time 150 ms, JR-delay 52 µs
II	^*1*^*H,*^*13*^*C-HSQC*^*BMRZ*^ Aromatic region (Bodenhausen and Ruben 1980)	800 MHz, ns: 8, sw(f2): 8.6 ppm, sw(f1): 24.8 ppm, aq(f2): 75 ms, aq(f1): 38 ms, o1(^1^H): 4.7 ppm, o2(^13^C): 143 ppm, o3(^15^N): 153 ppm, rel. delay: 1.0 s, time: 1 h	INEPT transfer time 2.7 ms (^1^J_CH_ 185 Hz), off-resonant Q3 shaped pulse for C5 decoupling at 95 ppm with 25 ppm bandwidth
III	^*1*^*H,*^*13*^*C-CT-HSQC*^*BMRZ*^ Full (Vuister and Bax 1992)	700 MHz, ns: 40, sw(f2): 10.0 ppm, sw(f1): 95.6 ppm, aq(f2): 73 ms, aq(f1): 17 ms, o1(^1^H): 4.7 ppm, o2(^13^C): 107 ppm, o3(^15^N): 153 ppm, rel. delay: 1.0 s, time: 7 h	INEPT transfer time 2.8 ms (^1^J_CH_ 180 Hz), CT period 25 ms (^1^J_CC_ 40 Hz)
IV	*(H)C(CCN)H*^*BMRZ*^ Aromatics-to-imino (Piotto et al. 1992; Sklenár et al. 1996; Wöhnert et al. 2003)	800 MHz, ns: 256, sw(f3): 20.9 ppm, sw(f2): 9.9 ppm, aq(f3): 67 ms, aq(f2): 32 ms, o1(^1^H): 4.7 ppm, o2(^13^C): 137 ppm, o3(^15^N): 154 ppm, rel. delay: 1.5 s, time: 16 h	CC-TOCSY mixing time 28 ms
V	*3D *^*13*^*C-detected CNC*^*BMRZ*^ C6/8-to-C1′ (Sklenar et al. 1993a)	800 MHz, ns: 24, sw(f3): 24.5 ppm, sw(f2): 34.7 ppm, sw(f1): 12.0 ppm, aq(f3): 67 ms, aq(f2): 6 ms, aq(f1): 20 ms, o1(^13^C): 90 ppm, o2(^1^H): 4.7 ppm, o3(^15^N): 157 ppm, rel. delay: 0.5 s, time: 48d h	C6/8-N1/9 transfer time 30 ms, C–H transfer time 2.9 ms (1′) and 2.6 ms (6/8)
VI	^*1*^*H,*^*1*^*H-TOCSY*^*KI*^ Excitation sculpting water suppression (Shaka et al. 1988; Shaka and Hwang 1995)	600 MHz, ns: 16, sw(f2): 8.8 ppm, sw(f1): 6.2 ppm, aq(f2): 100 ms, aq(f1): 51 ms, o1(^1^H): 4.7 ppm, o2(^13^C): 99 ppm, o3(^15^N): 86 ppm, rel. delay: 1.5 s, time: 3 h	CC-TOCSY mixing time 40 ms
VII	*3D *^*13*^*C-NOESY-HSQC*^*BMRZ*^ Aromatics (Piotto et al. 1992; Sklenar et al. 1993b)	800 MHz, ns: 16, sw(f3,^1^H): 8.8 ppm, sw(f2,^13^C): 22.0 ppm, sw(f1,^1^H): 6.2 ppm, aq(f3): 73 ms, aq(f2): 7 ms, aq(f1): 20 ms, o1(^1^H): 4.7 ppm, o2(^13^C): 142.5 ppm, o3(^15^N): 185 ppm, rel. delay: 1.0 s, time: 3 days 4 h	NOE mixing time 200 ms
VIII	*3D TROSY-HCCH-COSY*^*BMRZ*^ Adenine C2-to-C8 (Simon et al. 2001)	800 MHz, ns: 16, sw(f3,^1^H): 9.0 ppm, sw(f2,^13^C): 24.8 ppm, sw(f1,^13^C): 58.5 ppm, aq(f3): 71 ms, aq(f2): 6 ms, aq(f1): 5 ms, o1(^1^H): 4.7 ppm, o2(^13^C): 142.5 ppm, o3(^15^N): 150 ppm, rel. delay: 1.0 s, time: 1 day 19 h	Bruker standard parameter set
IX	*3D *^*13*^*C-NOESY-HSQC*^*BMRZ*^ Ribose (Piotto et al. 1992; Sklenar et al. 1993b)	600 MHz, ns: 16, sw(f3,^1^H): 10.4 ppm, sw(f2,^13^C): 43.0 ppm, sw(f1,^1^H): 8.3 ppm, aq(f3): 82 ms, aq(f2): 5 ms, aq(f1): 13 ms, o1(^1^H): 4.7 ppm, o2(^13^C): 82.5 ppm, o3(^31^P): − 1 ppm, rel. delay: 1.3 s, time: 2 days 10 h	NOE mixing time 200 ms, HSQC transfer time 2.9 ms (^1^J_CH_ 170 Hz)
X	*3D (H)CCH-TOCSY*^*BMRZ*^ ribose C1′-to-C2′ (Kay et al. 1993; Richter et al. 2010)	600 MHz, ns: 8, sw(f3,^1^H): 10.4 ppm, sw(f2,^13^C): 10.0 ppm, sw(f1,^13^C): 35.4 ppm, aq(f3): 82 ms, aq(f2): 21 ms, aq(f1): 12 ms, o1(^1^H): 4.7 ppm, o2(^13^C): 76.5 ppm, o3(^31^P): − 1 ppm, rel. delay: 1.3 s, time: 1 day 3 h	CC-TOCSY mixing time 6 ms
XI	*3D (H)CCH-TOCSY*^*BMRZ*^ ribose C1′-to-C5′ (Kay et al. 1993; Richter et al. 2010)	600 MHz, ns: 8, sw(f3,^1^H): 10.4 ppm, sw(f2,^13^C): 10.0 ppm, sw(f1,^13^C): 35.4 ppm, aq(f3): 82 ms, aq(f2): 21 ms, aq(f1): 12 ms, o1(^1^H): 4.7 ppm, o2(^13^C): 76.5 ppm, o3(^31^P): − 1 ppm, rel. delay: 1.3 s, time: 1 day 3 h	CC-TOCSY mixing time 18 ms
XII	*3D H(C)CH-TOCSY*^*BMRZ*^ Ribose C1′-to-H5′ (Kay et al. 1993; Richter et al. 2010)	700 MHz, ns: 16, sw(f3,^1^H): 8.6 ppm, sw(f2,^13^C): 10.0 ppm, sw(f1,^1^H): 3.2 ppm, aq(f3): 85 ms, aq(f2): 25 ms, aq(f1): 39 ms, o1(^1^H): 4.7 ppm, o2(^13^C): 76.5 ppm, o3(^15^N): 153 ppm, rel. delay: 1.0 s, time: 20 h	CC-TOCSY mixing time 16 ms

Experimental parameters and experiment-specific parameters are given

*ns* number of scans, *sw* spectral width, *aq* acquisition time, *o1/2/3* carrier frequencies on channels 1/2/3, *rel. delay* relaxation delay, *CT* constant time, *JR* jump-return

**Table 2:** List of NMR experiments conducted at KI and BMRZ at 283 K

#	NMR experiment	Experimental parameters	Characteristic parameters
I	^*1*^*H,*^*1*^*H-NOESY*^*KI*^ Jump-return water suppression	600 MHz, ns: 128, sw(f2): 21.0 ppm, sw(f1): 11.9 ppm, aq(f2): 81 ms, aq(f1): 25 ms, o1(^1^H): 4.7 ppm, o2(^13^C): 99 ppm, o3(^15^N): 153 ppm, rel. delay: 1.0 s, time: 16 h	NOE mixing time 150 ms, JR-delay 52 µs
II	*BEST-TROSY-HNN-COSY*^*BMRZ*^ Across hydrogen bond (Dingley and Grzesiek 1998; Wöhnert et al. 1999; Dingley et al. 2008)	600 MHz, ns: 64, sw(f3): 21.5 ppm, sw(f1): 98.9 ppm, aq(f3): 60 ms, aq(f1): 10 ms, o1(^1^H): 4.7 ppm, o2(^13^C): 140 ppm, o3(^15^N): 184 ppm, rel. delay: 0.3 s, time: 1.5 h	NN-transfer time 30 ms
III	^*1*^*H,*^*13*^*C-HSQC*^*BMRZ*^ Aromatic region (Bodenhausen and Ruben 1980)	950 MHz, ns: 4, sw(f2): 8.6 ppm, sw(f1): 26.1 ppm, aq(f2): 63 ms, aq(f1): 31 ms, o1(^1^H): 4.7 ppm, o2(^13^C): 143 ppm, o3(^15^N): 153 ppm, rel. delay: 1.0 s, time: 0.5 h	INEPT transfer time 2.7 ms (^1^J_CH_ 185 Hz), off-resonant Q3 shaped pulse for C5 decoupling at 95 ppm with 25 ppm bandwidth
IV	*H(N)CO*^*BMRZ*^ Imino-to-carbon (Favier and Brutscher 2011; Solyom et al. 2013)	950 MHz, ns: 32, sw(f3): 21.0 ppm, sw(f1): 27.9 ppm, aq(f3): 60 ms, aq(f1): 10 ms, o1(^1^H): 4.7 ppm, o2(^13^C): 157.5 ppm, o3(^15^N): 153 ppm, rel. delay: 0.3 s, time: 0.5 h	NC-INEPT transfer time 18 ms (^1^J_CN_ 28 Hz)
V	*(H)C(CCN)H*^*BMRZ*^ Aromatics-to-imino (Piotto et al. 1992; Sklenár et al. 1996; Wöhnert et al. 2003)	700 MHz, ns: 288, sw(f3): 23.0 ppm, sw(f2): 10.0 ppm, aq(f3): 70 ms, aq(f2): 36 ms, o1(^1^H): 4.7 ppm, o2(^13^C): 137 ppm, o3(^15^N): 154 ppm, rel. delay: 1.8 s, time: 21 h	CC-TOCSY mixing time 28 ms

Experimental parameters and experiment-specific parameters are given

*ns* number of scans, *sw* spectral width, *aq* acquisition time, *o1/2/3* carrier frequencies on channels 1/2/3, *rel. delay* relaxation delay, *JR* jump-return

### The U-rich bulge

One of the structural features of the SL5a RNA is an asymmetric U-rich bulge (Fig. 1c). In this likely more dynamic part of the RNA, a near to complete sequential walk (H6/8 to H6/8 or H1′) was possible and thus, all aromatic H6/8–C6/8 correlations were assigned. With the aromatic assignment at hand, the strong imino resonance of a uridine involved in non-canonical base pairing was assigned to residue U194 using the (H)C(CCN)H experiment at 283 K. From observation of this signal, the formation of a base pairing involving U194 and likely either U211 or U212 is suggested. This is further supported by an imino-to-imino NOE contact between U194 and a non-canonical uridine at 273 K. Furthermore, from the U194 carbon chemical shifts in the HNCO experiment, we conclude that the hydrogen bonding interaction is mediated through the C2 carbonyl group (Fürtig et al. 2003; Ohlenschläger et al. 2004). The existence of a GU- wobble base pair involving residues U195 and G210 has not been confirmed, yet. However, broadened imino proton resonances for an additional guanosine and uridine, which are taking part in non-canonical interactions, are observed at low temperature (283 K).

### The 5′-UUUCGU-3′ hexaloop

**Fig. 2:**
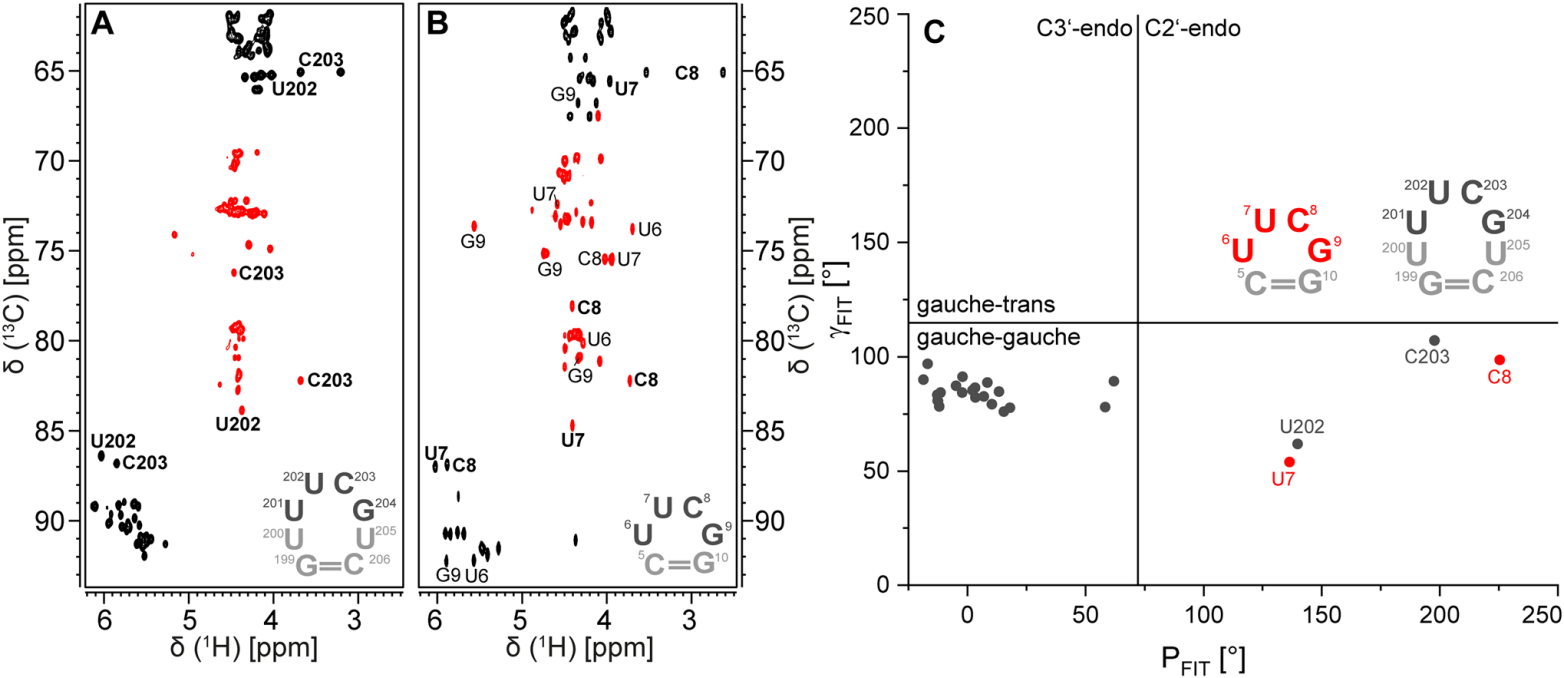
Comparison of ^1^H,^13^C-CT-HSQC spectra of the ribose regions of **a** SL5a and **b** a 14 nt RNA with 5′-cUUCGg-3′ tetraloop (Fürtig et al. 2004; Nozinovic et al. 2010). Positive contours are given in black, negative contours in red. Experimental details are given in Table 1. The loop sequences are displayed and U202/U7 and C203/C8 resonances are highlighted in bold font. **c** Canonical coordinates for all residues of SL5a with a complete carbon ribose assignment. For comparison, the canonical coordinates of residues U7 and C8 of a 14 nt RNA with 5′-UUCG-3′ tetraloop are given in red

In addition to the U-rich asymmetric bulge (Fig. 1c), SL5a features a 5′-UUUCGU-3′ hexaloop, which also caps the helix of SL5b in the 5′-UTR. Except for residue U205, all aromatic loop assignments were derived from sequential NOE correlations, e.g. H6/8 to H5 or H1′ to H6/8 sequential contacts. Since the central residues of this loop sequence, 5′-UUCG-3′, resemble a highly abundant and well-characterized tetraloop sequence (Cheong et al. 1990; Fürtig et al. 2004; Nozinovic et al. 2010), we asked, whether structural features of this UUCG tetraloop are also found within the 5′-UUUCGU-3′ hexaloop of SL5a. While the characteristic imino proton resonances of the sheared GU base pair in the 5′-UUCG-3′ tetraloop remained elusive in SL5a spectra (e.g. ^1^H 1D or ^1^H,^15^N-HSQC), ^1^H,^13^C-HSQC spectra of the ribose region of SL5a and a 14 nt RNA with a 5′-cUUCGg-3′ tetraloop (secondary structure Suppl. Figure 1B) yielded a similar peak pattern (Fig. 2a and b). Here, it is evident that the chemical shifts of the central two nucleotides of the 5′-UUUCGU-3′ hexaloop, U202 and C203, are in good agreement with the respective counterparts in the 5′-cUUCGg-3′ tetraloop. This observation is also reflected in the canonical coordinates (Ebrahimi et al. 2001; Cherepanov et al. 2010), which suggest the ribofuranosyl ring to adopt the C2′-endo conformation for U202 and C203, while the remaining nucleotides (with a complete ribose carbon assignment) adopt the canonical C3′-endo conformation (Fig. 2c). These spectral data suggest a structural similarity between the middle part of the 5′-UUUCGU-3′ hexa- and 5′-cUUCGg-3′ tetraloop. This might not hold true to the same extent for the flanking residues U201 and G204 as characteristic resonances are absent in the ^1^H,^13^C-HSQC spectrum of the ribose region (Fig. 2a and b). Thus, the detailed loop architecture remains subject to further structural investigation.

## Assignment and data deposition

The nearly complete resonance assignment of SL5a builds on the imino resonance assignment published earlier (Wacker et al. 2020). Starting from this assignment, all 33 aromatic H6–C6 and H8–C8 correlations were unambiguously assigned. Furthermore, the H2–C2 correlations of the two adenosines present in this RNA as well as all of the H5–C5 correlations of the uridines and cytidines were unambiguously assigned. In addition, the quaternary carbon atoms of the nucleobases in purines (C2: 77%, C4: 69%, C5: 62% and C6: 92%) and pyrimidines (C2: 15% and C4: 15%) were partially assigned. Here, uridine C2 and C4 resonances as well as guanosine C2 and G_-1_, G188, G198 and G208 C6 resonances were assigned at 283 K. Also, non-protonated tertiary nitrogen atoms of purines (N3: 15% (only adenosines assigned), N7: 100% and N9: 100%) and pyrimidines (N1: 95% and N3: 80% (cytidines)) were successfully assigned to a large extent. Within the ribose moieties, 91% of the H1′ and 91% of the C1′ atoms were assigned. Within the remaining ribose carbon atoms C2′–C5′, 77% were assigned. In summary, we assigned 97% of the ^1^H (H6/8, H5, H2, H1′) and 92% of the ^13^C (C6/8, C5(pyr), C1′) atoms, which are considered most important for an in-depth structural characterization. We updated the BMRB deposition with code 50346.

## Supplementary Information

Below is the link to the electronic supplementary material.

Supplementary file1 (DOCX 20962 KB)
