# Supplementary material for: 1H, 13C and 15N chemical shift assignment of the stem-loop 5a from the 5′-UTR of SARS-CoV-2
Source: Biomol NMR Assign. 2021 Jan 23;15(1):203–11. doi: 10.1007/s12104-021-10007-w (PMC7822759; doi:10.1007/s12104-021-10007-w)
Supplement: Supplementary file 1 — Supplementary file1 (DOCX 20962 KB) [file 12104_2021_10007_MOESM1_ESM.docx]

**Supplementary Information: ^1^H, ^13^C and ^15^N chemical shift assignment of the stem-loop 5a from the 5'-UTR of SARS-CoV-2**

Robbin Schnieders^2,4§^, Stephen A. Peter^1§^_,_ Elnaz Banijamali^6^, Magdalena Riad^6^, Nadide Altincekic^2,4^, Jasleen Kaur Bains^2,4^, Betül Ceylan^2,4^, Boris Fürtig^2,4^, J. Tassilo Grün^2,4^, Martin Hengesbach^2,4^, Katharina F. Hohmann^2,4^, Daniel Hymon^2,4^, Bozana Knezic^2,4^, Andreas Oxenfarth^2,4^, Katja Petzold^6^, Nusrat S. Qureshi^5^, Christian Richter^2,4^, Judith Schlagnitweit^6^, Andreas Schlundt^3,4^, Harald Schwalbe^2,4,*^, Elke Stirnal^2,4^, Alexey Sudakov^2,4^, Jennifer Vögele^3,4^ Anna Wacker^2,4^, Julia E. Weigand^1^, Julia Wirmer-Bartoschek^2,4^, Jens Wöhnert^3,4^

^1^Department of Biology, Technical University of Darmstadt, Schnittspahnstr. 10, 64287 Darmstadt, Germany. ^2^Institute for Organic Chemistry and Chemical Biology, ^3^Institute for Molecular Biosciences, ^4^Center for Biomolecular Magnetic Resonance (BMRZ), Johann Wolfgang Goethe-University Frankfurt, Max-von-Laue-Str. 7, 60438 Frankfurt/M., Germany.
^5^present address: EMBL Heidelberg, Meyerhofstraße 1, 69117 Heidelberg.
^6^Department of Medical Biochemistry and Biophysics, Karolinska Institute, Biomedicum, Solnavägen 9, 17177 Stockholm, Sweden

*to whom correspondence should be addressed: [covid19-nmr@dlist.server.uni-frankfurt.de](mailto:covid19-nmr@dlist.server.uni-frankfurt.de) , Schwalbe@nmr.uni-frankfurt.de

^§^ equal contribution

**Suppl. Table 1**: ^2h^J_NN_ coupling constants for the base pairs in SL5a. The coupling constants were derived at 283 K from 2D BEST-TROSY-HNN-COSY spectra.

| **base pair** | **^2h^J_NN_ coupling constant** |
| --- | --- |
| G188-C218 | 5.9 Hz |
| G189-C217 | 6.1 Hz |
| C190-G216 | 6.0 Hz |
| U191-A215 | 6.4 Hz |
| G192-C214 | 5.9 Hz |
| C193-G213 | 6.0 Hz |
| A196-U209 | 6.4 Hz |
| G198-C207 | 6.1 Hz |

**Suppl. Figure 1**: **A** Secondary structure of SCoV-2 SL5 with the Nsp1 coding region highlighted in orange. **B** Secondary structure of the 14 nt RNA with 5’-cUUCGg-3’ tetraloop (Fürtig et al. 2004; Nozinovic et al. 2010).


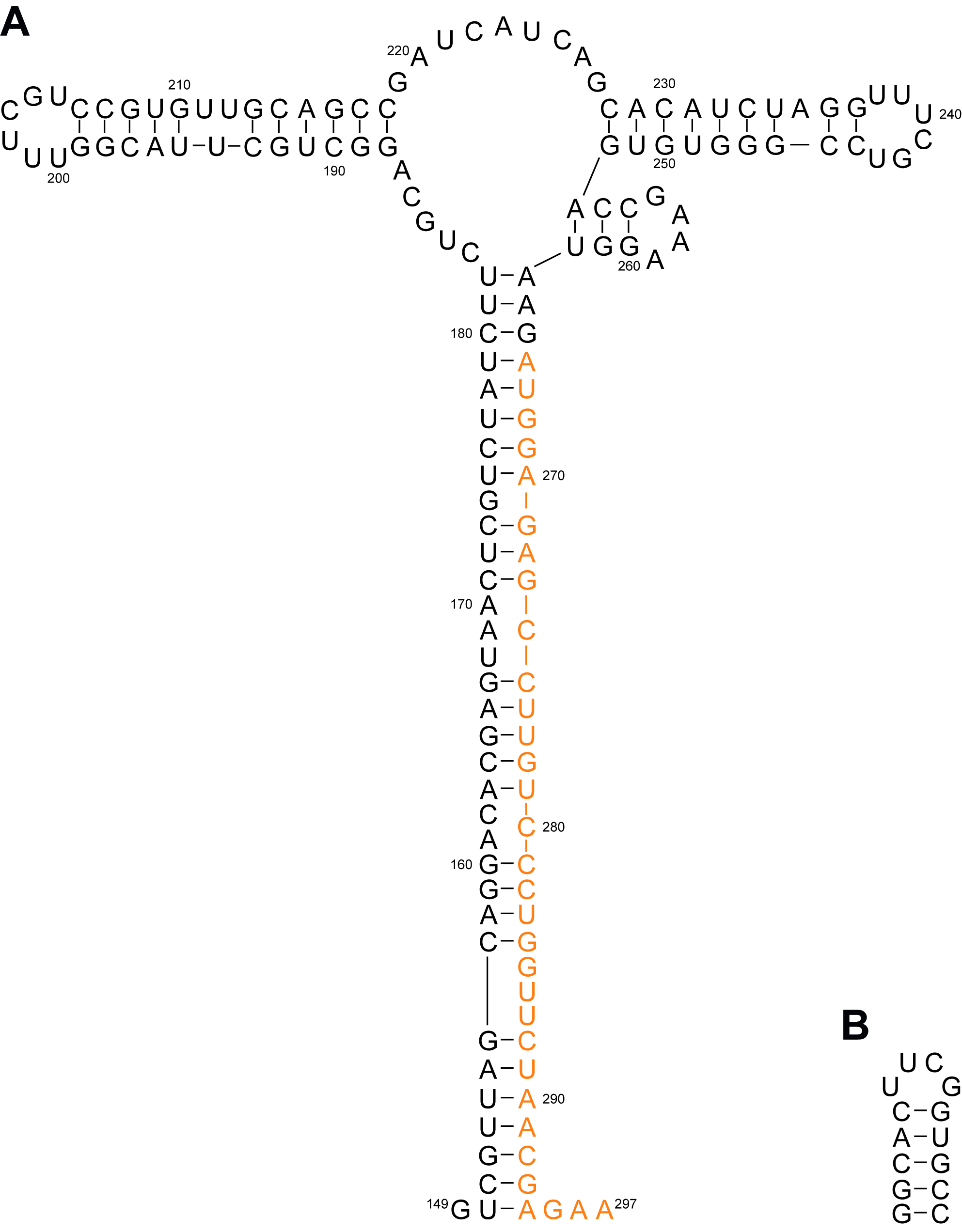


**References**

Fürtig B, Richter C, Bermel W, Schwalbe H (2004) New NMR experiments for RNA nucleobase resonance assignment and chemical shift analysis of an RNA UUCG tetraloop. J Biomol NMR 28:69–79 . https://doi.org/10.1023/B:JNMR.0000012863.63522.1f

Nozinovic S, Fürtig B, Jonker HRA, Richter C, Schwalbe H (2010) High-resolution NMR structure of an RNA model system: the 14-mer cUUCGg tetraloop hairpin RNA. Nucleic Acids Res 38:683–94 . https://doi.org/10.1093/nar/gkp956
